# Sensitivity of levofloxacin in combination with ampicillin-sulbactam and tigecycline against multidrug-resistant *Acinetobacter baumannii*

**Published:** 2017-02

**Authors:** Nahid Madadi-Goli, Rezvan Moniri, Sareh Bagheri-Josheghani, Nilufar Dasteh-Goli

**Affiliations:** 1 Department of Microbiology and Immunology, Faculty of Medicine, Kashan University of Medical Sciences, Kashan, Iran; 2 University of Maryland University College, Adelphi, Prince George's County, Maryland, USA

**Keywords:** *Acinetobacter baumannii*, Etest methods, Microbial drug resistance, Synergistic effect

## Abstract

**Background and Objectives:**

The selection of alternative treatment options with antibiotic combinations may be used for successful managing of multidrug-resistant *Acinetobacter baumannii*. The aim of this study was to determine the synergistic effects of ampicillin-sulbactam combined with either levofloxacin or tigecycline against MDR *A. baumannii.*

**Materials and Methods:**

A total 124 of *A.baumannii* isolates collected from clinical samples of hospitalized patients which assessed for antibiotic susceptibility using disk diffusion method. E-test was used on 10 MDR *A. baumannii* isolates to determine the minimum inhibitory concentration (MIC) of ampicillin-sulbactam, levofloxacin and tigecycline. Any synergistic effects were evaluated at their own MIC using E-test assay at 37°C for 24 hours. Synergy was defined as a fractional inhibitory concentration index (FICI) of ≤0.5.

**Results:**

Levofloxacin plus ampicillin-sulbactam combination was found to have synergistic effects (FIC index: ≤0.5) in 90% of the isolates, but there was no synergistic effect for ampicillin-sulbactam/tigecycline and tigecycline/levofloxacin combination. The antagonist effect in 50% of isolates (FIC index: >2) showed in combination of levofloxacin/tigecycline.

**Conclusion:**

The emergence of multidrug *A. baumannii* isolates requires evaluating by combination therapy. The combination of levofloxacin plus a bactericidal antibiotic such as ampicillin-sulbactam is recommended. Results should be confirmed by clinical studies.

## INTRODUCTION

During the last decades, emergence of antibiotic resistance among *A. baumannii* has been considered as a worldwide alarm. Importance to search new antimicrobial agents and/or therapeutic strategies has been a major challenge in the field of infectious diseases. Multidrug-resistant (MDR) *A. baumannii* is one of the main causes of severe hospital-acquired infections (1). Although, there are various effective antibacterial drugs, but currently polymyxin B and colistin, regardless of their adverse side effects, are only remaining options for treatment and control of infections caused by this pathogen (2, 3). The combination use of antibiotics has been shown to decreases the necessary therapeutic doses of antibacterial agents, which lower their risk of adverse reactions and drug toxicity (4).

Sulbactam is a beta-lactamase inhibitor that inhibits the activity of some plasmid-mediated beta-lactamases (5). Combination of sulbactam with ampicillin results to widened spectrum of activity against organisms with plasmid-mediated beta-lactamases.

Tigecycline inhibits bacterial protein synthesis by acting on the 30S ribosomal subunit and prevents amino acids to incorporate in process of elongating peptide chains (6). Tigecycline is a bacteriostatic and it has extended post antibiotic effect, which shows broad spectrum activity against Gram-negative organisms. The decreased susceptibility of Gram-negative organisms to tigecycline mostly mediated by theirs multidrug efflux pumps (7). Levofloxacin is a broad-spectrum bactericidal antibiotic that is active against Gram-negative bacteria. Its function mediated by inhibiting the DNA gyrase and topoisomerase IV. Unfortunately, the therapeutic choices for extensively drug-resistant (XDR) *Acinetobacter* are usually limited to polymyxins and tigecycline. Empiric antibiotic therapy for *A. baumannii* should be chosen based on local susceptibility patterns. It should be containing a broad-spectrum cephalosporin, a combination including sulbactam, or a carbapenem. A fluoroquinolone, an aminoglycoside, or colistin is second agent alternatives. The selection of alternative treatment options with antibiotic combinations may be used for successful managing of MDR *A. baumannii* infections. The aim of the present study was to perform *in vitro* research, using the eplisometer method; to investigate the efficacy of tigecycline combined with either levofloxacin or ampicillin-sulbactam of MDR *A. baumannii* isolates.

## MATERIALS AND METHODS

### Bacterial strains and antibiotic susceptibility

The antibiotic susceptibility test of 124 isolates was done using Kirby-Bauer disk diffusion on Mueller-Hinton agar plates, and the results were interpreted according to the Clinical and Laboratory Standards Institute (CLSI) criteria (8). The antibacterial disks were purchased from MAST, Merseyside, UK were as follow: piperacillin (100μg), ampicillin-sulbactam (10/10μg), piperacillin-tazobactam (100/10μg), cefotaxime (30μg), ceftazidime (30μg), ceftriaxone (30μg), imipenem (10μg), meropenem (10μg), gentamicin (10μg), amikacin (30μg), tetracycline (30μg), ciprofloxacin (5μg), levofloxacin (5μg), trimetho-prim-sulfamethoxazole (1.25/23.75mg); polymyxin B (300 IU) and colistin (10μg). *Escherichia coli* ATCC 25922 were used as quality control in each susceptibility test. Multi-drug resistance (MDR) is defined as resistance to three or more classes of antibiotics.

All isolates were subjected to PCR to determine the presence of *bla*_oxa-51_ gene, which is specific to *A. baumannii*. They were verified by blast analysis in the database (http://www.ncbi.nlm.nih.gov/GenBank). Primer pairs used for *bla*_OXA-51_ gene with 353 base pair chain shown as follows: Forward: 5'-TAATGCTTTGATCGGCCTTG-3', and Reverse: 5'-TGGATTGCACTTCATCTTGG-3'. *bla*_OXA-51_ PCR product was sequenced by Macrogen Company (Seoul, Korea), and the alignments were prepared with the Chromas 1.7.5 software and BLAST in NCBI. *A. baumannii* ATCC 19606 was used as reference strain. Minimum inhibitory concentrations (MICs) of levofloxacin, ampicillin-sulbactam, and tigecycline were obtained by E-test according to CLSI recommendations. Interpretation criteria for susceptibility tests were based on CLSI guidelines (8).

### Synergy testing.

For this study, 10 multidrug-resistant clinical isolates of *A. baumannii* were selected. Preparing fresh passages of the bacterial suspensions performed the synergy tests. The amount of 100μl bacterial suspensions prepared with 0.5 Mac-Farl and of standard turbidity, and was spread onto 150mm Muller–Hinton agar plates. E-test® strips (Liofilchem, Roseto Degli Abruzzi, Italy), for ampicillin/sulbactam and tigecycline were placed separately onto the plates. The exact site at which the strip was placed was marked on the plate. Then the plates were incubated for just 1h at 37°C. Following incubation, the E-test® strips were aseptically removed from the plates and the E-test® strip for levofloxacin was placed onto the marked space, accurately overlapping the first strip. Plates were then incubated for 24h at 37°C and the MIC values were recorded. The MIC was interpreted as the value at which the inhibition zone intersected the scale on the E strip. The obtained MIC results were converted to qualitative categories (susceptible, intermediate, and resistant) using CLSI guidelines (8), and compared with *Pseudomonas aeruginosa* ATCC 27853, as reference strain, was used as a quality control strain. Levofloxacin MIC (≤ 2 μg/ml sensitive, 4μg/ml intermediate, ≥8μg/ml resistant); ampicillin/sulbactam (≤ 8/4μg/ml sensitive, 16/8μg/ml intermediate, ≥32/16μg/ml resistant); tigecycline (≤ 2μg/ml sensitive, 4μg/ml intermediate, ≥8 μg/ml resistant).

### Fractional inhibitory concentration (FIC).

To evaluate the effect of the combination, the fractional inhibitory concentration (FIC) was calculated for each antibiotic in each combination as follows:

FIC index=FIC of drug A+FIC of drug B; where FIC of drugA=MIC of drug A in combination/MIC of drug A alone; and FIC of drug B=MIC of drug B in combination/MIC of drug B alone. An FIC index of ≤0.5 was defined as synergism, FIC index of 0.51–0.99 defined as additive effect, FIC index of 1–2 defined as indifference. The FIC index of >2 defined as antagonism (9).

## RESULTS

The phenotypic resistance patterns represented that the isolates are resistant to cefepime, cefotaxime, ceftazidime, ceftriaxone, ciprofloxacin, levofloxacin, piperacillin, and susceptible to colistin and polymyxin B (Table 1). *In vitro* antibacterial activity of tested antibiotics combinations against MDR *A. baumannii* isolates shown in Table 2, Table 3, and Table 4. Synergism was seen using levofloxacin plus ampicilin/sulbactam combination in 90% of tested isolates (Table 5). Antagonistic effect was seen in 50% of isolates with tigecycline plus levofloxacin combination. In this study, the combination of tigecycline plus ampicilin/sulbactam and tigecycline plus levofloxacin combination did not observe *in vitro* synergy. Indifferent effect was observed in 70% with combination of ampicilin/sulbactam plus tigecycline. The combination of tigecycline with levofloxacin demonstrated 20% additive and 30% indifferent effect against MDR *A. baumannii* isolates.

**Table 1.** Summary of resistance: phenotypic characteristics of the ten multidrug resistance *A. baumannii* isolates selected for synergy test

**Strain No.**	**PRL**	**SAM**	**PTZ**	**CAZ**	**CPM**	**CTX**	**CRO**	**MEM**	**IMI**	**AK**	**GM**	**LEV**	**CIP**	**T**	**TS**	**CO**	**PB**
1	R	R	R	R	R	R	R	R	R	S	R	R	R	R	R	S	S
2	R	R	R	R	R	R	R	R	R	R	R	R	R	R	R	S	S
3	R	R	R	R	R	R	R	R	R	R	R	R	R	R	R	S	S
4	R	R	R	R	R	R	R	R	R	S	R	R	R	R	R	S	S
5	R	R	R	R	R	R	R	R	R	R	R	R	R	R	R	S	S
6	R	S	R	R	R	R	R	S	S	S	I	R	R	S	I	S	S
7	R	S	R	R	R	R	R	R	S	S	R	R	R	R	R	S	S
8	R	R	R	R	R	R	R	R	R	R	R	R	R	R	R	S	S
9	R	S	S	R	R	R	R	S	S	S	I	R	R	R	R	S	S
10	R	R	R	R	R	R	R	R	R	I	R	R	R	I	R	S	S

R: Resistance S: Sensitive I: Intermediate

PRL: Piperacillin; SAM: Ampicillin-sulbactam; PTZ: Piperacillin-tazobactam; CAZ: Ceftazidime; CPM: Cefepime; CTX: Cefotaxime; CRO: Ceftriaxone; MEM: Meropenem; IMI: Imipenem; AK: Amikacine; GM: Gentamicin; LEV: Levofloxacin; CIP: Ciprofloxacin; T: Tetracycline, TS: Trimethoprim-sulfamethoxazole; CO: Colistin; PB: Polymyxin B

**Table 2.** *In vitro* antibacterial activity of ampicilin/sulbactam with levofloxacin combinations against MDR *A.baumannii* isolates

**Bacterial strain number**	**MIC (alone) Levofloxacin MIC μg/ml**	**MIC (alone) ampicilin/sulbactam MIC μg/ml**	**MIC combined ampicilin/sulbactam plus Levofloxacin MIC μg/ml**	**FIC index**	**Interaction**
1	32	32	4	0.25	Synergism
2	32	16	4	0.375	Synergism
3	32	32	4	0.25	Synergism
4	16	32	3	0.28	Synergism
5	32	32	6	0.375	Synergism
6	32	6	0.75	0.148	Synergism
7	32	24	4	0.291	Synergism
8	32	2	4	2.125	Antagonist
9	32	6	0.75	0.148	Synergism
10	32	32	6	0.375	Synergism

**Table 3.** *In vitro* antibacterial activity of levofloxacin with tigecycline combinations against MDR *A. baumannii* isolates

**Bacterial strain number**	**MIC (alone) Levofloxacin MIC μg/ml**	**MIC (alone) Tigecycline MIC μg/ml**	**MIC combined Levofloxacin plustigecycline MIC μg/ml**	**FIC index**	**Interaction**
1	32	4	12	3.375	Antagonism
2	32	12	12	1.375	Indifference
3	32	16	8	0.75	Additive
4	16	0.75	0.75	1.05	Indifference
5	32	16	12	1.125	Indifference
6	32	1.5	4	2.79	Antagonism
7	32	4	8	2.25	Antagonism
8	32	2	12	6.375	Antagonism
9	32	16	8	0.75	Additive
10	32	4	12	3.375	Antagonism

**Table 4.** *In vitro* antibacterial activity of ampicilin/sulbactam with tigecycline combinations against ten MDR *A. baumannii* isolates

**Bacterial strain number**	**MIC (alone) ampicilin/sulbactam MIC μg/ml**	**MIC (alone) tigecycline MIC μg/ml**	**MIC combined ampicilin/sulbactam plustigecycline MIC μg/ml**	**FIC index**	**Interaction**
1	32	8	12	1.875	Indifference
2	24	16	12	1.25	Indifference
3	24	16	24	2.5	Antagonism
4	24	1	0.75	0.78	Additive
5	32	16	16	1.5	Indifference
6	8	4	3	1.125	Indifference
7	24	12	12	1.5	Indifference
8	4	2	1	0.75	Additive
9	32	16	12	1.125	Indifference
10	32	8	12	1.875	Indifference

**Table 5.** Synergy test results for ampicillin-sulbactam/tigecycline, levofloxacin/ampicillin-sulbactam and tigecycline/levofloxacin combination against ten MDR *A. baumannii* isolates

**Effect Combination**	**Synergistic No. (%)**	**Indifferent No. (%)**	**Antagonostic No. (%)**	**Additive No. (%)**
Ampicillin-sulbactam/tigecycline	0(0)	7(70)	1(10)	2(20)
Levofloxacin/ampicillin-sulbactam	9(90)	1(10)	0(0)	0(0)
Tigecycline/levofloxacin	0(0)	3(30)	5(50)	2(20)

## DISCUSSION

The emergence of antibiotic resistance *A. baumannii* is increasing at an alarming rate all around the world and especially in Iran (10–16). Data from the surveillance of antimicrobial resistance studies in Iran showed high resistance rate to ceftazidime, imipenem, or meropenem (11, 13–14). MDR *A. baumannii* is a noticeable challenge in Iranian hospitals (13–16). *A. baumannii* has the ability to develop resistance through some various means, leading to emergence of global drug-resistant isolates, which are more complicated to treat and are related with a higher mortality rates than susceptible isolates. Prior carbapenems and fluoroquinolones exposure isolates are more related with colonization and unresponsiveness infections due to drug-resistant *A. baumannii* isolates.

In our setting with high rate of resistance rate to the broad-spectrum cephalosporin, a combination of beta-lactam/beta-lactamase inhibitor and carbapenem, therapeutic options is polymyxins and possibly tigecycline. Colistin is currently the treatment of choice for infections caused by MDR *A. baumannii*. However, colistin administration alone is also related with significant nephrotoxicity and hetero-resistance in MDR *A. baumannii* clinical isolates.

Peerayeh et al. had studied *in vitro* activity of tigecycline and colistin against clinical isolates of *A. baumannii* isolated in several hospitals in Tehran and Bandar-Abbas, Iran (3). According to their results all isolates were sensitive to colistin and polymyxin-B. Moreover, based on the FDA criteria, the resistance rates for tigecycline were 20.8% and 17.6% in Tehran and Bandar-Abbas, respectively (3). Tigecycline resistance is chiefly resulted from resistance-nodulation-cell division (RND)-type transporters, mostly the AdeABC, AdeFGH and AdeIJK efflux pumps, but other resistance mechanisms have also been concerned (17). When two antimicrobial agents act concurrently on a pathogen, their effects may be synergism, antagonism indifference, or addition. All of these effects may be observed both in-vitro and in-vivo. Those effects that can be attained with combinations of antimicrobial drugs may vary with different combinations and are specific for each kind of strain of microorganisms. For the antagonist effect, should be remembered that the combined action is less effective than that used alone.

According to our results ampicilin/sulbactam and levofloxacin showed highly synergism effect in MDR *A. baumannii* strains isolated from patients (defined as a fractional inhibitory concentration index of < 0.5). In this study, levofloxacin failed in vitro activity as a single agent against the 10 clinical isolates. Levofloxacin and Ampicilin/sulbactam resistance were the frequently identified isolates. However, levofloxacin and ampicilin/sulbactam combination showed a synergistic effect against MDR *A. baumannii* isolates and increased the antibiotic activity of each drug, suggesting that the combination may improve effects of both antibiotics and combat drug-resistant bacteria that cause MDR *A. baumannii* infections. The MIC values of the combination were reduced in relation to the MIC values of each levofloxacin alone (Table 2). These results produce promises that levofloxacin can be used as combination therapy for infections by MDR *A. baumannii.* Ampicilin/sulbactam and levofloxacin may apply different pharmacokinetic and pharmacodynamic properties under *in-vitro* and *in-vivo* conditions. Further work on the pharmacokinetic parameters of this combination in vivo will be needed. In this study antagonist effects observed in half of isolates when used combination of levofloxacin plus tigecycline which is defined as a fractional inhibitory concentration index of > 2. Antagonism effect occurred when a bacteriostatic drug was given with a bactericidal one (18). While tigecycline, a bacteriostatic agent is used in combination with levofloxacin, a bactericidal drug, and their effects should be antagonized. Principe et al. shown that all 24 *A. baumannii* isolates were resistant to levofloxacin and piperacillin-tazobactam. Chequer board analysis performed with all antimicrobials in combination with tigecycline showed 5.9% synergy, 85.7% indifference, and 8.3% antagonism. Tigecycline showed synergistic activity with levofloxacin, amikacin, imipenem and colistin. Particularly, synergistic effects were seen only among tigecycline resistant isolate. Antagonistic interactions were observed for only one isolates to tigecycline/ampicillin-sulbactam (19). The results of study of Petersen et al. on the interaction of tigecycline with 13 select antimicrobial agents against a wide variety of Gram-negative and Gram-positive isolates described that tigecycline in combination with ampicillin/sulbactam and levofloxacin resulted in no interaction or synergy. Antagonism was not seen for any combination (20). *In vitro* activities of levofloxacin in combination with tigecycline using a microbroth checkerboard technique showed that synergistic interaction for tigecycline-levofloxacin combinations (21).

The antagonism effect mainly occur if the bacteriostatic drug reaches the site of infection prior to bactericidal drug, or the killing of bacteria is essential for cure, and if only minimal effective doses of either drug in the pair are present. In addition, an unpredicted variety of cellular responses to antagonistic drug combinations are possible. It signifies that multiple mechanisms that cause the interactions. Control of multidrug-resistant *A. baumannii* is concerned as an important worldwide problem. More recently, emergence of extensively drug-resistant *A. baumannii* infections presents a significant challenge to global control. Prevention of drug-resistant *Acinetobacter* depends on early detection, control of spread, and preventing establishment of endemic strains by avoiding exposure of microorganisms to a particularly valuable drug by limiting its use, especially at hospitals. Selection of the suitable treatment for MDR strains of *A. baumannii* is critical in practice, because usual laboratory testing is unable to disclose the susceptibility of two agents from different classes of antibiotics. Most of the infectious diseases practitioners prefer to prescribe two agents from distinct classes of antibiotics to prevent emergence of drug resistance and treatment failure. Sometimes, the synergy testing revealing the potential effectiveness of the two antibiotics in combination, but it must be confirmed by the patient’s response during clinical trials. In the other hand, in spite of sensitivity of the infectious agents against MDR *A. baumannii*, to 2 tested antibiotics in synergy testing, their use in patients may not thoroughly cure the infection, and it seems need to do more investigations. In combination therapy for MDR *A. baumannii*, the infectious diseases clinician should avoid recommending two antibiotics, one from bactericidal class and the other from bacteriostatics. A limitation of this study is the use of inadequate isolates.

## CONCLUSION

In the present study, all of the isolated MDR *A. baumannii* were resistant to all tested antibiotics except to colistin and polymixin B. It is an important alarming sign that represents a major epidemiological health concern and limits the therapeutic choices in critically ill patients with MDR *A. baumannii* infections. Ampicilin/sulbactam combined with levofloxacin revealed synergistic effect in the most MDR *A. baumannii* isolates. The synergistic effect did not observe in levofloxacin/tigecycline, and ampicilin-sulbactam/tigecycline combination. According to the results of this study the combination of levofloxacin plus a bactericidal antibiotic such as ampicillin-sulbactam are recommended.

## References

[1] Zilberberg MD Kollef MH Shorr AF Secular trends in *Acinetobacter baumannii* resistance in respiratory and blood stream specimens in the United States, 2003 to 2012: A survey study. J Hosp Med 2015; doi: 10.1002/jhm.2477. 26353076

[2] Thamlikitkul V Tiengrim S Seenama C *In vitro* activity of polymyxin B against carbapenem-resistant *Acinetobacter baumannii*. J Med Assoc Thai 2014; 97: 1254– 1258. 25764631

[3] Peerayeh SN Karmostaji A Sarasiabi SS Javadpour S Davoodian P Moradi N In Vitro Activity of Tigecycline and Colistin against clinical isolates of *Acinetobacter baumannii* in Hospitals in Tehran and Bandar-Abbas, Iran. Electron Physician 2014; 1; 6: 919– 924. 25763168 10.14661/2014.919-924 PMC4324296

[4] Berditsch M Jäger T Strempel N Schwartz T Overhage J Ulrich AS Synergistic effect of membrane-active peptides polymyxin B and gramicidin S on multidrug-resistant strains and biofilms of *Pseudomonas aeruginosa*. Antimicrob Agents Chemother 2015; 59: 5288– 5296. 26077259 10.1128/AAC.00682-15 PMC4538509

[5] Bush LM Johnson CC Ureidopenicillins and beta-lactam/beta-lactamase inhibitor combinations. Infect Dis Clin North Am 2000; 14: 409– 433. 10829263 10.1016/s0891-5520(05)70255-5

[6] Jean SS Hsieh TC Hsu CW Lee WS Bai KJ Lam C Comparison of the clinical efficacy between tigecycline plus extended-infusion imipenem and sulbactam plus imipenem against ventilator-associated pneumonia with pneumonic extensively drug-resistant *Acinetobacter baumannii* bacteremia, and correlation of clinical efficacy with in vitro synergy tests. J Microbiol Immunol Infect 2016; 49: 924– 933. 26341302 10.1016/j.jmii.2015.06.009

[7] Peleg AY Adams J Paterson DL Tigecycline Efflux as a Mechanism for Nonsusceptibility in *Acinetobacter baumannii*. Antimicrob Agents Chemother 2007; 51: 2065– 2069. 17420217 10.1128/AAC.01198-06 PMC1891386

[8] Performance standards for antimicrobial susceptibility testing, “Twenty third Information Supplement. 2013 M100–S23. p. 66.

[9] Sopirala MM Mangino JE Gebreyes WA Biller B Bannerman T Balada-Llasat JM Synergy testing by Etest, microdilution checkerboard, and time-kill methods for pan-drug-resistant *Acinetobacter baumannii*. Antimicrob Agents Chemother 2010; 54: 4678– 4683. 20713678 10.1128/AAC.00497-10 PMC2976112

[10] Lowings M Ehlers MM Dreyer AW Kock MM High prevalence of oxacillinases in clinical multidrug-resistant *Acinetobacter baumannii* isolates from the Tshwane region, South Africa - an update. BMC Infect Dis 2015; 15: 521. 26573617 10.1186/s12879-015-1246-8 PMC4647659

[11] Nageeb W Metwally L Kamel M Zakaria S In vitro antimicrobial synergy studies of carbapenem-resistant *Acinetobacter baumannii* isolated from intensive care units of a tertiary care hospital in Egypt. J Infect Public Health 2015; 8: 593– 602. 26116454 10.1016/j.jiph.2015.05.007

[12] Van TD Dinh QD Vu PD Nguyen TV Pham CV Dao TT Antibiotic susceptibility and molecular epidemiology of *Acinetobacter calcoaceticus-baumannii* complex strains isolated from a referral hospital in northern Vietnam. J Glob Antimicrob Resist 2014; 2: 318– 321. 25540720 10.1016/j.jgar.2014.05.003 PMC4270437

[13] Ghajavand H Esfahani BN Havaei SA Moghim S Fazeli H Molecular identification of *Acinetobacter baumannii* isolated from intensive care units and their antimicrobial resistance patterns. Adv Biomed Res 2015; 4: 110. 26261812 10.4103/2277-9175.157826 PMC4513333

[14] Kooti S Motamedifar M Sarvari J Antibiotic resistance profile and distribution of oxacillinase genes among clinical isolates of *Acinetobacter baumannii* in Shiraz teaching hospitals, 2012–2013. Jundishapur J Microbiol 2015; 8( 8): e20215. 26464764 10.5812/jjm.20215v2 PMC4600599

[15] Sharif F Moniri R Bagheri Josheghani S Dastehgoli K Antibiotic susceptibility patterns and *bla*_PER-1_ β-lactamase-producing *Acinetobacter baumannii* isolated from hospitalized patients. J Glob Antimicrob Resist 2015; 3: 47– 48. 27873651 10.1016/j.jgar.2014.11.002

[16] Pajand O Rezaee MA Nahaei MR Mahdian R Aghazadeh M Soroush MH Study of the carbapenem resistance mechanisms in clinical isolates of *Acinetobacter baumannii*: comparison of burn and non-burn strains. Burns 2013; 39: 1414– 1419. 23726475 10.1016/j.burns.2013.03.024

[17] Pournaras S Koumaki V Gennimata V Kouskouni E Tsakris A In Vitro Activity of Tigecycline Against *Acinetobacte rbaumannii*: Global Epidemiology and Resistance Mechanisms. Adv Exp Med Biol 2016; 897: 1– 14. doi: 10.1007/5584-2015-5001. 26563303

[18] Ocampo PS Lázár V Papp B Arnoldini M Abel zurWiesch P Busa-Fekete R Antagonism between bacteriostatic and bactericidal antibiotics is prevalent. Antimicrob Agents Chemother 2014; 58: 4573– 4582. 24867991 10.1128/AAC.02463-14 PMC4135978

[19] Principe L D’Arezzo S Capone A Petrosillo N Visca P In vitro activity of tigecycline in combination with various antimicrobials against multidr ug resistant *Acinetobacter baumannii*. Ann Clin Microbiol Antimicrob 2009 21; 8: 18. 19460166 10.1186/1476-0711-8-18 PMC2693502

[20] Petersen PJ Labthavikul P Jones CH Bradford PA *In vitro* antibacterial activities of tigecycline in combination with other antimicrobial agents determined by chequerboard and time-kill kinetic analysis. J Antimicrob Chemother 2006; 57: 573– 576. 16431863 10.1093/jac/dki477

[21] Ozbek B Sentürk A Postantibiotic effects of tigecycline, colistin sulfate, and levofloxacin alone or tigecycline-colistin sulfate and tigecycline-levofloxacin combinations against *Acinetobacter baumannii*. Chemotherapy 2010; 56: 466– 471. 21088399 10.1159/000321015

